# Lipocalin-2 as a marker of inflammation, bone density, and triglyceride-glucose index for new-onset arthritis patients in Mosul, Iraq

**DOI:** 10.5339/qmj.2024.23

**Published:** 2024-05-06

**Authors:** Safa Rabea Saadon, Thikra Ali Allwsh

**Affiliations:** 1 Department of Chemistry, Collage of Science, University of Mosul, Mosul, Iraq *Email: thekraaliallwsh@uomosul.edu.iq

**Keywords:** Lipocalin-2, arthritis, inflammation, vitamin D3, Triglyceride Glucose Index, Mosul, Iraq

## Abstract

Objective: Lipocalin-2 is an acute phase-associated adipokine that can serve as an inflammatory and biomarker indicator of cartilage deterioration in osteoarthritis. However, its role in the musculoskeletal system remains not fully understood. Hence, this study aimed to evaluate lipocalin-2 and its relationship with markers of inflammation (Interferon-gamma, ESR, and CRP), bone density (vitamin D3 and calcium), and the triglyceride-glucose index in new-onset arthritis patients in Mosul, Iraq.

Methods: This study included 125 participants aged 20 to 65, divided into two groups. The Arthritis Patient Group comprised 70 participants (37 females and 33 males) attending the Bone Diseases Consultation Unit at the Ibn Sina Teaching Hospital in Mosul, Iraq. The Control Group comprised 31 females and 24 males. Ethical approval was obtained from the Iraqi Ministry of Health - Nineveh Health (No. 2022095).

Commercial ELISA kits were used to measure serum lipocalin-2, Interferon-gamma, ESR, and CRP as inflammation markers, vitamin D3, and calcium as bone density markers. Moreover, the Triglyceride Glucose (TYG) Index was evaluated.

Results: The findings revealed a significant increase in lipocalin-2 levels in males compared to females, with LCN-2 increasing with age. Arthritis patients showed a significant increase (72%) in lipocalin-2 levels. Inflammatory indicators (erythrocyte sedimentation rate, C-reactive protein, interferon-gamma) displayed significant increases (46%, 1200%, and 581%, respectively). Glucose (23%), triglycerides (71%), and TYG index (21%) also exhibited significant increases. Meanwhile, bone density indicators (vitamin D3 and calcium) found a significant decrease (53% and 20%, respectively) in arthritis patients.

Linear correlation coefficient (R) analysis revealed a significant positive relationship between lipocalin-2 and indicators of inflammation, glucose, TG, and TYG index.

Conclusion: This study’s findings suggest that LCN-2 serum levels were higher in patients with new-onset arthritis than in controls in Mosul, and LCN-2 serum increased in males compared with females and getting older serum LCN-2 increased for the patients and control groups. Furthermore, a significant correlation was found between the Triglyceride Glucose Index, which measures metabolic disorders, and serum LCN-2 levels and inflammatory indicators in new-onset arthritis patients in Mosul, Iraq.

## Introduction

Arthritis is a complex and common medical illness characterized by stiffness and inflammation in one or more bodily joints.^1^ Arthritis is associated with inflammation, and even though the pathogenic process of the various forms varies,2 it affects the synovial joints and the tissues around them and causes a musculoskeletal system disorder. Most signs of this condition are pain, stiffness, impairment, loss of joint mobility, and the potential for long-term disability, which is directly tied to age.^3^ It also affects all age groups, including children, and the incidence in women is higher than.^4^ Arthritis has a greater impact than just the pain it causes to individuals since it has a significant financial and medical cost impact. Early identification of high-risk groups based on risk factors and the development of efficient management strategies are essential for both people and society, given the rising incidence of arthritis and related medical expenses.^5^

Lipocalin-2 (LCN-2) belongs to the Lipocalins family, which is known as a group of proteins that play an important role in cellular transport, as it works to transport hydrophobic molecules such as fatty acids, prostaglandins, retinoids, and steroid hormones to the target organs, in addition to its ability to transport iron.^6,7^ It is expressed in joint tissues and is stimulated by inflammatory mediators as it is a mechanically responsive adipokine. In osteoblasts, the absence of mechanical loading leads to increased expression of LCN-2, which appears to contribute to bone metabolism by stimulating pro-osteogenic factors, nuclear factor kappa receptor activator, receptor activator of nuclear factor kappa-B ligand (RANKL) and interleukin-6 (IL-6).^8^

A study showed that the level of LCN-2 was increased in patients with psoriasis and rheumatoid arthritis.^9^ It has also been found that serum LCN-2 levels can be used as an indicator of structural damage in rheumatoid arthritis (RA).^10^ In another study, LCN-2 levels were shown to be greater in RA patients compared to osteoarthritis individuals and normal controls.^11^ However, there is a need to study serum LCN-2 levels in patients with new-onset arthritis.

The C-reactive protein (CRP) and the erythrocyte sedimentation rate (ESR) are inflammatory markers elevated in the acute phase of arthritis. CRP is also an immune regulator that has an important function in pathways of inflammation associated with atherogenic.^12^

IFN-γ plays an essential role in bone and cartilage homeostasis, which mediates immune and inflammatory responses and has been found to play a role in the pathogenesis of joint diseases and, thus, joint deterioration.^13,14^ Studies have shown the relationship between interferon-gamma to stimulate expression of lipocalin-2 in adipose tissue and the effect of its Intraperitoneal injection for treatment and activation on the STAT1 pathway.^7,15^ Accordingly, this study aimed to estimate the level of interferon-gamma in the serum of new-onset arthritis and to find its relationship to serum LCN-2 as an inflammatory marker.

Vitamin D reduces the incidence of fractures and slows down bone loss in elderly persons. In addition to calcium, vitamin D aids in treating and preventing osteoporosis. A vitamin D deficit reduces calcium absorption, which causes calcium release from the bones.^16^

Furthermore, it has been shown glucose has direct effects on cartilage metabolism, promoting the synthesis of inflammatory mediators that can contribute to cartilage degradation and arthritis.^17^ A novel marker that has gained popularity is the TYG index, which can be used to evaluate metabolic dysfunction and insulin resistance.^18^ The TYG index is appealing because it makes use of easily accessible clinical data. Numerous studies have shown that the TYG index can be used as a surrogate marker to identify people who may be at risk for metabolic diseases such as type 2 diabetes, nonalcoholic fatty liver disease, and coronary artery disease.^19^ The TYG index is a potentially useful tool for evaluating various disorders due to its ease of use and potential clinical value.^20^ The relationship of the triglyceride-glucose (TyG) index to new arthritis is one of the recent topics. A recent article found the (TyG) index is considered a marker for predicting new arthritis in people aged more than 45.^21^

The current study aimed to clarify the role of lipocalin in Mosul patients with new-onset arthritis by assessing its association with indicators of inflammation (IFN-γ, CRP, and ESR), bone density (vitamin D3 and calcium), and metabolic dysfunction (triglyceride-glucose (TyG) index).

## Materials and Methods

### Study Design

This research was a case-control study for the control and patient group:

### Arthritis Patient Group

Inclusion of 70 participants with arthritis, 37 were females and 33 males (ages 20–65 years), all arthritis patients of Arab origins from the city of Mosul, Iraq, visiting the Ibn Sina Teaching Hospital, noting that the patients were diagnosed by specialized doctors and were chosen at random. Patients’ information was recorded according to the questionnaire paper, noting that the patients had no family history of the disease.

### The Control Group

Fifty-five participants of healthy individuals, which included 31 females and 24 males, matched the patient’s age and body mass index for comparison with the arthritis patient group. They do not have any medical conditions.

### Exclusion Criteria

Subjects with diabetes mellitus, high blood pressure, pregnant women, liver diseases, and any other known case of inflammation were excluded from the present study.

### Ethical Approval

This study (No. 2022095) received ethical approval from the Iraqi Ministry of Health - Nineveh Health on 5/7/2022. Consent was acquired from each participant.

### Measurement of Demographic and Biochemical Parameters

Preparation of Sample: (5 ml) of venous blood was drawn after an overnight fast [of 12 hours] from all participants, and blood was centrifuged for 10-15 minutes at 3500 rpm to get the serum.

### Lipocalin-2 and Interferon-gamma Were Measured Using an Enzyme-Linked

Immunosorbent Assay (ELISA) kit from the SUN LONG Biological Technology Co., Ltd kit (China). The evaluation was done according to the instructions provided by the manufacturer.

The Erythrocyte Sedimentation Rate (ESR) was estimated using the Westergren method, which depends on the sedimentation rate of red blood cells. Vitamin D3 was measured using an electrochemiluminescence (ECL) kit with Cobas e411 analyzers. Also, calcium was estimated using a BIOSYSTEM kit (Spain), and C-reactive protein (CRP) was estimated using a Plasmatic kit (France).

The glucose and triglyceride (TG) concentration was estimated using ready-made assay (kits) from the company BIOLABS and enzymatic methods. All the parameters for the kits were evaluated according to the instructions provided by the manufacturer. The triglyceride Glucose (TYG) Index was calculated using the following:^22^

(TYG) = In [TG (mg/dl) × fasting glucose (mg/dl) /2]

### Data Collection

A methodical coding procedure was applied to data obtained from clinical exams, laboratory studies, and outcome measurements. After being coded, the data was later organized and entered into Microsoft Excel.

### Data Analysis

The data is shown as mean ± SE. The comparison between the arthritis patient group and the control group using the t-test. Pearson correlation coefficient (r) was applied to determine the relation between parameters based on linear regression analysis. P values less than or equal to 0.05 are considered significant.

## Results

### The Serum Lipocalin-2 (LCN-2) in the New-onset Arthritis Patients

The results in Table 1 showed a significant increase in the lipocalin-2 concentration in males compared with females for the patients and control group. Also, the results showed a significant increase (72%) in the total mean concentration of serum LCN-2 in the new-onset arthritis patients compared to its concentration in the control group in Mosul at a level (P ≤ 0.01).

The results in Table 2 showed a significant increase in the concentration of lipocalin in the age group (46-65 years) compared to the age group (20-45 years) in the two groups of patients and healthy control at a level (P ≤ 0.01). So, notice that as age increases, serum LCN-2 increases.

### Inflammatory Markers Concentration

The results in Table 3 showed a significant increase (46%) in the concentration of interferon-gamma (INF-γ) in the serum of the arthritis patient group compared to its concentration in the control group at a level (P ≤ 0.01). Also showed a significant increase (1200%) in the concentration of C- Reactive Protein (CRP) and (581%) sedimentation rate of red blood cells (Erythrocyte Sedimentation Rate (ESR)) at the level (P ≤ 0.001) in the serum of arthritis patient group compared to the control group.

### Bone Density Marker Concentration

A significant decrease (53%) was found in the concentration of vitamin D3 in serum of the arthritis patient group compared to its concentration in the control group at a level (P ≤ 0.01) as well, a significant decrease (20%) was found in the concentration of calcium at the level (P ≤ 0.05) in serum of the arthritis patient group compared to its concentration in serum of the control group, as shown in Table 4.

### Glucose Triglyceride Index (TYG index)

The results shown in Table 5 showed a significant increase (23%) in the concentration of fasting glucose in the serum of the arthritis patient group compared to its concentration in the control group at the level (P ≤ 0.01). The results also showed a significant increase (71%) in the concentration of Triglyceride (TG) in the serum of the arthritis patient group, as well as a significant increase (21%) in the Glucose triglyceride index (TYG Index) in the arthritis patient group at the level of (P ≤ 0.05) compared to the control group.

### Linear Correlation Between Lipocalin-2 and Clinical Variables

Table 6 showed a positive significant correlation between lipocalin-2 and both CRP and ESR at the level (P ≤ 0.01). Also, a positive significant correlation was found between lipocalin-2 and glucose, TG, and the TYG index at the level (P ≤ 0.01) in both the control group and the arthritis patient group. At the same time, there was no significant correlation between lipocalin-2 and interferon-gamma (INF-γ), vitamin D3, and calcium.

## Discussion

Based on this study results, it appears that the LCN-2 serum of patients with new-onset arthritis was greater than controls in Mosul. This is consistent with Sarhat et al., which found an increase in LCN-2 in rheumatoid arthritis patients and increased LCN-2 in patients with psoriasis. It became clear that LCN-2 increased more in rheumatoid arthritis compared to osteoarthritis individuals and healthy.^9,11,23^ However, we investigated the literature and found no studies evaluating the relationship between LCN-2 levels and new-onset arthritis, especially in Mosul, Iraq.

Lipocalin-2 is secreted in response to inflammation, stimulates its secretion by pro-inflammatory cytokines, and is associated with many markers of inflammation. Also, increased lipocalin-2 is correlated with severity and indicates an active disease state.^24^ According to these, lipocalin-2 might be a reliable indicator of acute inflammatory response in arthritis.

The increase in lipocalin-2 with age may be because of a decrease in muscle mass and an increase in fat mass due to physical inactivity and weight gain associated with aging, as adipose tissue activates macrophages and secretes adipokines and cytokines that stimulate the secretion of lipocalin-2. The increase in lipocalin-2 in males compared to females may be attributed to the regulation of lipocalin-2, which is affected by hormonal factors.

A high concentration of INF-γ was found in patients, which may be attributed to the fact that INF-γ is a cytokine secreted by T cells in response to the immune and inflammatory systems and linked with rheumatoid arthritis.^25^ Also, C-reactive protein (CRP) is a sensitive marker of systemic inflammation in arthritis, where a high CRP was found in patients. The reason is that CRP is a protein produced by liver cells, and its concentration increases during inflammatory processes, where it is stimulated by pro-inflammatory cytokines, especially interleukin-6, which is the cytokine that causes arthritis.^26^ The patients also displayed a high ESR level, where its elevation indicates the presence of inflammation in the body, as inflammatory conditions lead to an increase in the number of proteins in the blood plasma. These proteins work to adhere red blood cells to each other and increase the speed of their sedimentation.^27^

The decrease in vitamin and calcium may be because vitamin D is responsible for regulating calcium and maintaining a healthy skeleton and may decrease in arthritis patients due to lack of physical activity in the open air and lack of exposure to sunlight, as well as due to not consuming it in food or the presence of factors that reduce its absorption.^28,29^ There is an inverse relationship between pro-inflammatory cytokines and vitamin D, as individuals with low vitamin D concentrations are more susceptible to arthritis. This correlation aligns with elevated glucose because cytokines affect metabolic processes, causing high glucose.^30,31^ Additionally, research showed that pro-inflammatory cytokines work to disrupt the insulin signaling pathway, contributing to the high TG levels due to the relationship with the distribution of adipose tissue. It was found that the increase in the mass of adipose tissue is responsible for bearing the increase in the abnormal burden on the joints, and it also has a role in the production of adipokines that are part of the inflammatory mechanism.^32,33^

The results showed an increase in the glucose triglyceride index (TYG Index) in new-onset arthritis patients in Mosul, and this is consistent with Liu et al. that the (TyG) index is considered a marker for predicting new arthritis and the TYG Index is a new and easy measure of insulin resistance and inflammation.^1,22,34^

The positive correlation between lipocalin-2 and glucose, TG, and TYG index in both the control group and arthritis patient group is due to the positive correlation between lipocalin-2 secretion in adipose tissue and its concentration in the blood with signs of insulin resistance, hyperglycemia, and levels of glucose transporters. It has been demonstrated that the skeleton controls glucose and energy metabolism. LCN-2, which is released by osteoblasts, reduces fat mass, and inhibits hunger while enhancing glucose metabolism.^35^ It might be a treatment for obesity and metabolic syndrome.^36^ Also, the positive correlation between lipocalin-2 CRP and ESR is attributed to the fact that they are considered important inflammatory indicators, and their production is stimulated by pro-inflammatory cytokines that play an important role in causing arthritis.^12,37^ Lipocalin-2 promotes neutrophils to release pro-inflammatory cytokines such as interleukins, tumor necrosis factor (TNF-α), and nuclear factor kappa B (NF-kB) proteins are activated as well as LCN2 levels have been associated with an increased inflammatory environment of bone. Consequently, serious illnesses may result from neutrophil malfunction.

## Conclusion

This study demonstrated, by the results, that it appears that levels of LCN-2 serum of patients with new-onset arthritis were greater than controls in Mosul, and LCN-2 serum increased in males compared with females and getting older serum LCN-2 increased for the patients and control group. A significant correlation was found between serum LCN-2 levels and CRP and ESR, as well as with the TYG index. To the best of our knowledge, this association has not been assessed previously. Therefore, it can be considered a new indicator and a promising tool for diagnosing initiation. Also, understanding the mechanisms will make establishing new diagnosing, preventive, and treatment methods easier.

## Acknowledgment

The authors would like to thank the Nineveh Health/Ibn Sina Teaching Hospital in Mosul and the University of Mosul for providing all the required facilities set up for the current research work. Also, the authors sincerely thank the patients for their active participation in this study project, which greatly aided in its success.

## Authors’ Contributions

Each author of this manuscript contributed equally and significantly to the research. After a group review, they gave their approval to the manuscript’s final draft.

## Conflicts of Interest

The authors declare no conflict of interest.

## Availability of Data

The corresponding author can provide the datasets used and/or analyzed in this study upon reasonable request.

## Figures and Tables

**Table 1.** Lipocalin-2 concentration in the arthritis and control group.

	**Lipocalin-2 (ng/ml)**		
**Group**	**Females Mean ± SD (No.)**	**Males Mean ± SD (No.)**	**Total (125)**
**Control**	61.88 ± 13.7	82.36 ± 15.69	72.71 ± 15.52
Mean ± SD (No.)	(31)	(24)	(55)
**Arthritis Patient**	117.07 ± 18.1*	132.53 ± 14.45*	124.38 ± 23.58*
Mean ± SD (No.)	(37)	(33)	(70)
%	+ 87	+ 60	+ 73

* Significant at the level P ≤ 0.01.

+ increase concentration.

**Table 2.** The effect of age on Lipocalin-2 concentration in the arthritis and control group.

	**Lipocalin-2 ng/ml**	
**Age (year)**	**Control Group**	**Arthritis Patient Group**
	Mean ± SD	Mean ± SD
20-45	57.9 ± 11.6	111.4 ± 11.01
46-65	85.8 ± 13.19*	139.5 ± 9.15*

* Significant at the level P ≤ 0.01.

**Table 3.** Inflammatory markers concentration in the control and arthritis patient groups.

	**Control Group**	**Arthritis Patient Group**		
**Inflammatory Markers**	**Mean ± SD**	**Mean ± SD**	**%**	**P-value**
interferon-gamma (ng/ml)	60.46 ± 11.64	88.9 ± 12.38	+46	0.01
C- Reactive Protein (mg/l)	3.25 ± 0.72	52 ± 8.37	+1200	0.001
Erythrocyte Sedimentation Rate (mm/h)	8.12 ± 2.73	55.09 ± 6.23	+581	0.001

+ increase concentration.

**Table 4.** Bone density marker concentration in the control and arthritis patient groups.

	**Control Group**	**Arthritis Patient Group**		
**Bone Density Marker**	**Mean ± SD**	**Mean ± SD**	**%**	**P-value**
Vitamin D3 (ng/ml)	42 ± 10.62	18.15 ± 4.65	−53	0.01
Calcium (mg/dl)	9.81 ± 1.8	7.41± 1.05	−20	0.05

− decrease concentration.

**Table 5.** Clinical variables concentration in the control and arthritis patient groups.

	**Control Group**	**Arthritis Patient Group**		
**Clinical Variables**	**Mean ± SD**	**Mean ± SD**	**%**	**P-value**
Glucose (G)(mg/dl)	90.1 ± 6.23	109.33 ± 13.07	+ 23	0.01
Triglyceride (TG)(mg/dl)	104.25 ± 16.57	180.33 ± 14.8	+ 71	0.01
TYG index	4.06 ± 0.08	4.93 ± 0.3	+ 21	0.05

+ increase concentration.

**Table 6.** Linear correlation between lipocalin-2 and clinical variables.

	**Lipocalin-2 ( ng/ml)**	
	**Arthritis Patient Group**	**Control Group**
**Clinical Variables**	**R-value**	**R-value**
interferon-gamma	+0.233	+0.398
Erythrocyte Sedimentation Rate	+0.490*	+0.360*
C- Reactive Protein	+0.512*	+0.341*
Vitamin D3	-0.614	-0.568
Calcium	-0.403	-0.422
Glucose mg/dl	+0.476*	+0.425*
Triglycerides	+0.364*	+0.334*
TYG	+0.278*	+0.240*

* Significant at the level P ≤ 0.01.

## References

[bib1] Yan Y, Zhou L, La R, Jiang M, Jiang D, Huang L, Xu W, Wu Q (2023). The association between triglyceride glucose index and arthritis: a population-based study. Lipids Health Dis.

[bib2] Spel L, Martinon F (2020). Inflammasomes contributing to inflammation in arthritis. Immunol Rev.

[bib3] Klimak M, Nims RJ, Pferdehirt L, Collins KH, Harasymowicz NS, Oswald SJ (2021). Immunoengineering the next generation of arthritis therapies. Acta Biomater.

[bib4] Semenistaja S, Skuja S, Kadisa A, Groma V (2023). Healthy and Osteoarthritis-Affected Joints Facing the Cellular Crosstalk. Int J Mol Sci.

[bib5] Safiri S, Kolahi AA, Hoy D, Buchbinder R, Mansournia MA, Bettampadi D (2020). Global, regional, and national burden of neck pain in the general population, 1990-2017: systematic analysis of the Global Burden of Disease Study 2017. BMJ.

[bib6] Asaf S, Maqsood F, Jalil J, Sarfraz Z, Sarfraz A, Mustafa S (2023). Lipocalin 2-not only a biomarker: a study of current literature and systematic findings of ongoing clinical trials. Immunol Res.

[bib7] Jaberi SA, Cohen A, D’Souza C, Abdulrazzaq YM, Ojha S, Bastaki S (2021). Lipocalin-2: Structure, function, distribution and role in metabolic disorders. Biomed Pharmacother.

[bib8] Tu C, He J, Wu B, Wang W, Li Z (2019). An extensive review regarding the adipokines in the pathogenesis and progression of osteoarthritis. Cytokine.

[bib9] Wang D, Fang L, Pan G (2019). Association of Serum Lipocalin-2 Concentrations with Psoriasis and Psoriatic Arthritis: An Updated Meta-Analysis. Dis Markers.

[bib10] Gulkesen A, Akgol G, Poyraz A, Aydin S, Denk A, Yildirim T (2017;). Lipocalin 2 as a clinical significance in rheumatoid arthritis. Central European Journal of Immunology.

[bib11] Katano M, Okamoto K, Arito M, Kawakami Y, Kurokawa MS, Suematsu N (2009;). Implication of granulocyte-macrophage colony-stimulating factor induced neutrophil gelatinase-associated lipocalin in pathogenesis of rheumatoid arthritis revealed by proteome analysis. Arthritis Res Ther.

[bib12] Cui S, Qian J (2023). Future Biomarkers for Infection and Inflammation in Rheumatoid Arthritis. J Inflamm Res.

[bib13] Tang M, Tian L, Luo G, Yu X (2018). Interferon-Gamma-Mediated Osteoimmunology. Front Immunol.

[bib14] Nees TA, Rosshirt N, Zhang JA, Reiner T, Sorbi R, Tripel E (2019). Synovial Cytokines Significantly Correlate with Osteoarthritis-Related Knee Pain and Disability: Inflammatory Mediators of Potential Clinical Relevance. J Clin Med.

[bib15] Zhao P, Elks CM, Stephens JM (2014). The induction of lipocalin-2 protein expression in vivo and in vitro. J Biol Chem.

[bib16] Laird E, Ward M, McSorley E, Strain JJ, Wallace J (2010). Vitamin D and bone health: potential mechanisms. Nutrients.

[bib17] Ribeiro M, López de Figueroa P, Blanco FJ, Mendes AF, Caramés B (2016). Insulin decreases autophagy and leads to cartilage degradation. Osteoarthritis Cartilage.

[bib18] Ramdas Nayak VK, Satheesh P, Shenoy MT, Kalra S (2022). Triglyceride Glucose (TyG) Index: A surrogate biomarker of insulin resistance. J Pak Med Assoc.

[bib19] Xu L, Wu M, Chen S, Yang Y, Wang Y, Wu S (2022). Triglyceride-glucose index associates with incident heart failure: A cohort study. Diabetes Metab.

[bib20] Wang Z, Qian H, Zhong S, Gu T, Xu M, Yang Q (2023). The relationship between triglyceride-glucose index and albuminuria in United States adults. Front Endocrinol (Lausanne).

[bib21] Liu Y, Yao J, Xue X, Lv Y, Guo S, Wei P (2024). Triglyceride-glucose index in the prediction of new-onset arthritis in the general population aged over 45: the first longitudinal evidence from CHARLS. Lipids Health Dis.

[bib22] Zheng Y, Yin G, Chen F, Lin L, Chen Y (2022). Evaluation of Triglyceride Glucose Index and Homeostasis Model of Insulin Resistance in Patients with Polycystic Ovary Syndrome. Int J Womens Health.

[bib23] Sarhat ER, Alddin Ibrahim SK (2020;). Assessment of Serum Levels of Fetuin-A, Lipocalin-2, Interleukin-18, and C-Reactive Protein in Rheumatoid Arthritis Patients: A Biochemical Study. Cellular and Molecular Science.

[bib24] De la Chesnaye E, Manuel-Apolinar L, Oviedo-de Anda N, Revilla-Monsalve MC, Islas-Andrade S (2016). Las diferencias de género en los niveles plasmáticos de la lipocalina 2 se correlacionan con la edad y con la proporción de triglicéridos y lipoproteínas de alta densidad en individuos sanos [Gender differences in lipocalin 2 plasmatic levels are correlated with age and the triglyceride/high-density lipoprotein ratio in healthy individuals]. Gac Med Mex.

[bib25] Kato M (2020). New insights into IFN-γ in rheumatoid arthritis: role in the era of JAK inhibitors. Immunol Med.

[bib26] Shrivastava AK, Singh HV, Raizada A, Singh SK, Pandey A, Singh N (2015). Inflammatory markers in patients with rheumatoid arthritis. Allergol Immunopathol (Madr).

[bib27] Ajadi RA, Adebiyi AA, Otesile EB, Kasali OB (2018). Erythrocyte Sedimentation Rates and Leukogram Changes in Canine Model of Osteoarthritis. Niger J Physiol Sci.

[bib28] Charoenngam N (2021). Vitamin D and Rheumatic Diseases: A Review of Clinical Evidence. Int J Mol Sci.

[bib29] Aslam MM, John P, Bhatti A, Jahangir S, Kamboh MI (2019). Vitamin D as a Principal Factor in Mediating Rheumatoid Arthritis-Derived Immune Response. Biomed Res Int.

[bib30] Tudorachi NB, Eva I, Dascalu CG, Al-Hiary R, Barbieru B, Paunica M (2020;). The influence of serum calcium and magnesium levels in the radiological evolution of knee osteoarthritis. Journal of Mind and Medical Sciences.

[bib31] Wang CR, Tsai HW (2022). Immediate-release tofacitinib reduces insulin resistance in non-diabetic active rheumatoid arthritis patients: A single-center retrospective study. World J Diabetes.

[bib32] Salem HR, Zahran ES (2021). Vascular cell adhesion molecule-1 in rheumatoid arthritis patients: Relation to disease activity, oxidative stress, and systemic inflammation. Saudi Med J.

[bib33] Neumann E, Hasseli R, Ohl S, Lange U, Frommer KW, Müller-Ladner U (2021). Adipokines and Autoimmunity in Inflammatory Arthritis. Cells.

[bib34] Zheng Y, Rajcsanyi LS, Kowalczyk M, Giuranna J, Herpertz-Dahlmann B, Seitz J (2023). Lipocalin 2 - mutation screen and serum levels in patients with anorexia nervosa or obesity and in lean individuals. Front Endocrinol (Lausanne).

[bib35] Mosialou I, Shikhel S, Luo N, Petropoulou PI, Panitsas K, Bisikirska B (2020). Lipocalin-2 counteracts metabolic dysregulation in obesity and diabetes. J Exp Med.

[bib36] Mera P, Ferron M, Mosialou I (2018). Regulation of Energy Metabolism by Bone-Derived Hormones. Cold Spring Harb Perspect Med.

[bib37] Caliri AW, Tommasi S, Besaratinia A (2021). Relationships among smoking, oxidative stress, inflammation, macromolecular damage, and cancer. Mutat Res Rev Mutat Res.

